# Knowledge mapping of prodromal Parkinson’s disease: A bibliometric review and analysis (2000–2023)

**DOI:** 10.1097/MD.0000000000036985

**Published:** 2023-02-02

**Authors:** Shun Wang, Ning An, Yulin Wang, Yuan Li, Hailong Li, Yan Bai

**Affiliations:** a Department of Acupuncture and Moxibustion, The First Affiliated Hospital of Heilongjiang University of Traditional Chinese Medicine, Harbin, China; b Second Clinical Medical College, Heilongjiang University of Traditional Chinese Medicine, Heilongjiang, China; c Department of Science and Technology, Heilongjiang University of Traditional Chinese Medicine, Heilongjiang, China; d Department of Acupuncture and Moxibustion, The First Affiliated Hospital of Harbin Medical University, Heilongjiang, China; e Department of Acupuncture and Moxibustion, Heilongjiang Academy of Traditional Chinese Medicine, Heilongjiang, China; f Department of Acupuncture and Moxibustion, Heilongjiang Academy of Traditional Chinese Medicine, Institute of Acupuncture and Moxibustion, Heilongjiang, China.

**Keywords:** bibliometrics, CiteSpace, prodromal Parkinson’s disease, VOSviewers, Web of Science Core Collection

## Abstract

The prodromal period of Parkinson’s disease (PD) is currently a hot topic in PD research. However, no bibliometric analysis has been conducted in this research field. This study aimed to provide a comprehensive overview of the status, hotspots, and trends in the prodromal period of PD using bibliometrics. CiteSpace and visualization of similarities viewer were used to analyze articles and reviews on the prodromal period of PD in the Web of Science Core Collection (WoSCC) database. We analyzed the data on countries, institutions, journals, authors, keywords, and cited references. In total, 909 articles from 65 countries, including the United States (n = 265, 29.15%) and Germany (n = 174, 19.14%), were included. The number of articles and reviews related to the prodromal period of PD has increased yearly. The University of Tubingen (n = 45, 4.95%), McGill University (n = 33, 3.63%), and University of London (n = 33, 3.63%) were the research institutions with the most published studies. Movement Disorders is the journal with the largest number of published papers (n = 98, 10.8%) and the most cited publications (co-citation = 7035). These publications are from 4681 authors, with Berg (n = 49, 5.39%) and Postuma (n = 40, 4.40%) publishing the most publications, and Postuma’s study (n = 1206) having the most citations. Studying the nonmotor symptoms of PD precursors is a major topic in this research field. This is the first bibliometric study to comprehensively summarize the research trends and developments in the prodromal period of PD. This information identifies recent research frontiers and hotspots and provides a reference for scholars studying the prodromal period of PD.

## 1. Introduction

Parkinson’s disease (PD) is the second most common neurodegenerative disease worldwide, after Alzheimer’s disease, with a high incidence in middle-aged and elderly people. Despite extensive research on PD, its pathogenesis and underlying mechanisms remain unclear. At present, the gradual degeneration, necrosis, and apoptosis of dopaminergic neurons in the substantia nigra and striatum, and the continuous decrease in dopamine release in the substantia nigra-striatum pathway are considered the main pathological changes in this disease. Thus far, the development of Parkinson’s has been irreversible. All current drugs and surgical treatments can only improve clinical symptoms but cannot cure the disease, which significantly affects the physical and mental health of patients and places a huge burden on patients and their families. The current diagnostic criteria for PD are mainly motor symptoms, although 5–20 years before the onset of motor symptoms, dopamine (DA) neurons in the nigrostriatum disappear, and nonmotor symptoms appear. Nonmotor symptoms in the prodromal stage of PD mainly include olfactory disturbances, cognitive dysfunction, anxiety and depression, gastrointestinal dysfunction, and sleep disturbances.

At this time, the motor symptoms of PD are not yet apparent, and the onset is hidden. Thus, PD is easily ignored and misdiagnosed, leading to the loss of valuable treatment opportunities for patients. Therefore, early diagnosis, pathophysiological research, and treatment during the prodromal phase of PD are critical for timely and effective interventions in the progression of PD, which may slow or prevent the occurrence of motor symptoms. The International Parkinson and Movement Disorder Society (MDS) divides early PD into 3 stages: preclinical, involving only neurodegenerative changes without any associated symptoms; prodromal, in which symptoms and signs appear and persist but do not meet the clinical diagnostic criteria for PD; and clinical, in which motor symptoms exist and meet the clinical diagnostic criteria.^[1]^ The prodromal period of PD has become a popular research topic.

Bibliometric analysis is an important tool for comprehensively understanding research progress in a specific scientific data field. It can measure the interrelationships and influence of publications through a series of mathematical and statistical tools and has been implemented in many research fields. In this study, visualization of similarities viewer (VOSviewer) and CiteSpace were used to explore and visualize scientific publications on the prodromal phase of PD based on the Web of Science database. As no bibliometric analysis of the current situation, hot spots, and trends in the prodromal period of PD has been conducted, this study used VOSviewer and CiteSpace to address such a lack.

## 2. Methods

### 2.1. Data acquisition and retrieval strategy

**Figure 1.**
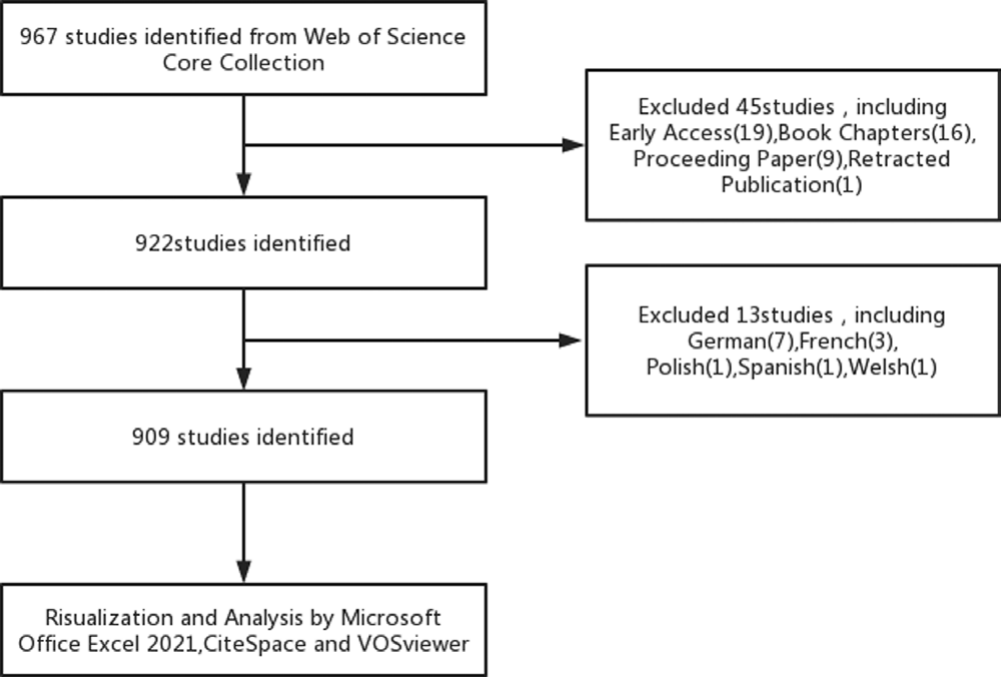
Publications screening flowchart.

We conducted a comprehensive literature search on the Web of Science Core Collection database (https://www.webofscience.com/wos/WoSCC/basic-search)on May 6, 2023. The search strategy employed the following formula: TS=(“Parkinson Disease”OR“Idiopathic Parkinson’s Disease”OR“Lewy Body Parkinson’s Disease”OR“Parkinson’s Disease,Idiopathic”OR“Parkinson’s Disease,Lewy Body”OR“Parkinson Disease,Idiopathic”OR“Parkinson’s Disease”OR“Idiopathic Parkinson Disease”OR“Lewy Body Parkinson Disease”OR“Primary Parkinsonism”OR“Parkinsonism,Primary”OR“Paralysis Agitans”)AND TS=(“Prodromal Symptoms”OR“Prodromal Symptom”OR“Symptom,Prodromal”OR“Symptoms,Prodromal”OR“Prodromal Syndromes”OR“Prodromal Syndrome”OR“Syndrome,Prodromal”OR“Syndromes,Prodroma l”OR“Prodromal Characteristics”OR“Characteristic,Prodromal”OR“Characteristics,Prodrom al”OR“Prodromal Characteristic”OR“Prodromal Signs”OR“Prodromal Sign”OR”Sign,Prodromal” OR”Signs,Prodromal”OR”ProdromaI States”OR”States,ProdromaI”OR”States,ProdromaI Period”OR”Periods,Prodromal”OR”Periods,Prodromal Periods”Or”Prodromal stage ”Or”Prodrom al stages”Or”stage,Prodromal”). The time range was set from January 1, 2000 to May 1, 2023. Only articles and reviews written in English were included for analysis. All selected records were exported in plain text format (Win UTF-8) and subsequently analyzed using CiteSpace (version 6.2.2; Drexel University, Philadelphia, Pennsylvania) and VOSviewer (version 1.6.19; Leiden University, Leiden, the Netherlands). Please refer to Figure 1 for an overview of the literature selection process.

### 2.2. Data analysis

VOSviewer (version 1.6.19) is a bibliometric analysis software that facilitates the extraction of crucial information from numerous publications.^[2]^ In our research, this software primarily undertakes the following analyses: country and institution; journal and co-cited journal analysis; author and co-cited author analysis; as well as co-cited reference analysis. The generated map in VOSviewer represents various entities such as countries, institutions, journals, and authors through nodes, where node size indicates quantity while color signifies the classification of these entities, respectively. Furthermore, the thickness of lines connecting nodes reflects the degree of collaboration or co-citation among these entities.CiteSpace (version 6.2.2), developed by Chen^[3]^, is another bibliometric analysis and visualization software.

In our study, CiteSpace was employed to generate bi-plot overlays, citation burst maps, and conduct keyword co-occurrence maps as well as keyword cluster analysis for journals. Additionally, the quantitative analysis of publications was performed using Microsoft Office Excel 2021.

## 3. Results

### 3.1. Quantitative Analysis of Publication

**Figure 2.**
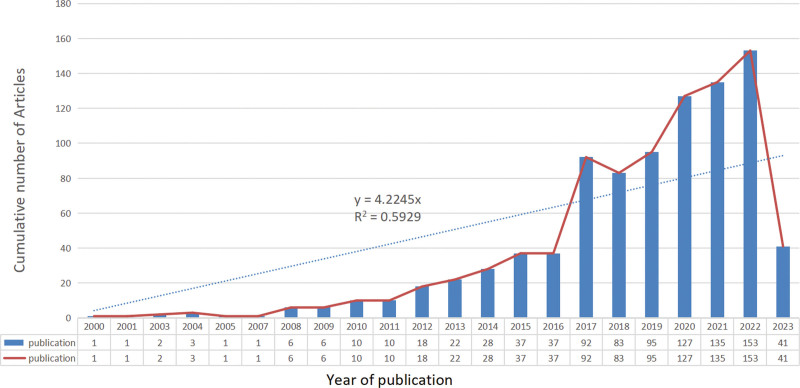
Number of annual publications.

According to our search strategy, a total of 909 studies on the prodromal period of PD were included in the bibliometric analysis from January 1, 2000 to May 1, 2023. These studies involved researchers from 65 countries/regions and affiliated with 1430 institutions. The findings were published in a diverse range of journals (273) and authored by a large number of contributors (4681 authors). Among these publications, there were 634 articles and 275 review articles. Based on the growth rate of annual publication volume, we divided the entire period into 3 distinct periods: the first period (2000–2007), the second period (2008–2016), and the third period (2017–2022). Please note that statistics for the year 2023 are not included in this analysis. As depicted in Figure 2, during the first period, only 9 papers were published with an average annual publication count of approximately 1.5. This indicates that research on the prodromal phase of PD was scarce at its early stage when it was still emerging as a field. In contrast, during the second period, there was a significant increase in publications with a total count of 174 papers and an average annual publication count of about19.3. This suggests that research on this topic entered its initial stage where more attention was given to understanding prodromal symptoms. The third period witnessed substantial growth in research output with a total count of 685 papers published. The average annual publication count reached around 114.2 papers per year during this rapid development stage. Revised sentence: “In 2017, a total of 92 papers were published, which was 2.49 times higher than the number in 2016”. By 2022, the publication count on the prodromal period of PD had reached 153. Furthermore, there has been a consistent annual increase in publications on this topic from 2017 to 2022. It should be noted that as of May 1, 2023, literature statistics for that year are incomplete; however, based on the fitting curve analysis, it is evident that the number of publications will continue to rise in subsequent years.

### 3.2. Contribution of countries/regions

**Figure 3.**
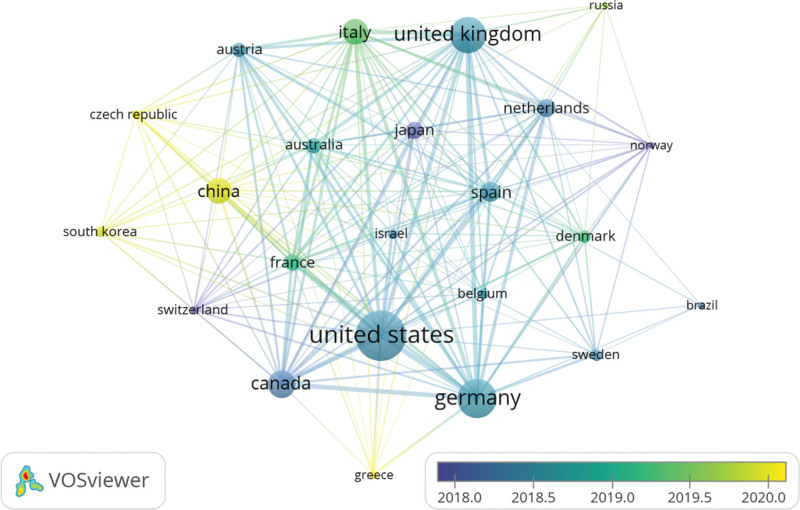
Cooperative network of countries.

A total of 62 countries contributed to the analysis of the prodromal period of PD in this study. The cooperation network by country is illustrated in Figure 3 using VOSviewer, and Table 1 lists the top 15 contributing countries. Among these, 10 are located in Europe (n = 10), 2 in Asia (n = 2), and 2 in North America (n = 2). The United States had the highest number of publications (n = 265, 29.15%), followed by Germany (n = 174, 19.14%) and the United Kingdom (n = 155, 17.05%). We filtered and visualized data from the top 23 countries with at least 10 publications to construct a collaborative network based on publication count and relationships within each country (Fig. 3). Furthermore, the United States exhibited the highest centrality value (0.33), suggesting its significant research relevance and collaboration with other countries. Notably, there was extensive active cooperation observed among different countries and regions; for instance, close collaborations were observed between the United States, Germany, Canada, and the United Kingdom.

**Table 1:** Top 15 productive countries/regions.

Ranking	Countries/regions	Counts	Percentage	citations	Centrality	Total link strength
1	United States	265	29.15	15346	0.33	325
2	Germany	174	19.14	10876	0.13	309
3	United Kingdom	155	17.05	12110	0.18	257
4	Canada	105	11.55	7889	0.07	212
5	Italy	95	10.45	3375	0.08	183
6	China	95	10.45	2168	0.04	72
7	Spain	65	7.15	4295	0.18	141
8	Netherlands	55	6.05	2994	0.05	107
9	Japan	51	5.61	1457	0.02	45
10	France	46	5.06	2546	0.08	142
11	Australia	43	4.73	5091	0.13	124
12	Austria	37	4.07	5582	0.01	113
13	Denmark	29	3.19	1232	0.06	54
14	Sweden	26	2.86	999	0.01	45
15	Belgium	24	2.64	706	0	43

### 3.3. Contribution of institutions

**Figure 4.**
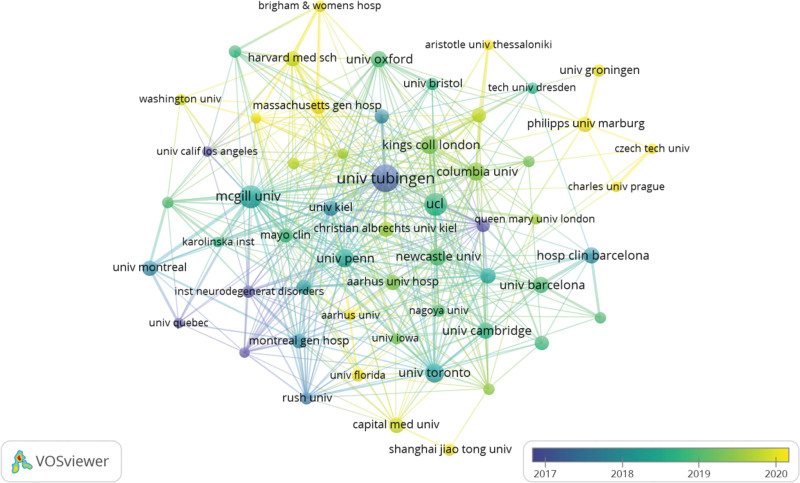
Cooperative network of institution analysis.

In this analysis, a total of 1430 institutions contributed to research on the prodromal phase of PD. Table 2 presents the top 15 institutions with the highest number of published papers. The most prolific institution was the University Tubingen (n = 45, 4.95%), followed by McGill University (n = 33, 3.63%) and University of London (UCL) (n = 33, 3.63%). We filtered and visualized data from 56 research institutions that had published at least 10 papers to generate an institutional collaboration network focused on the prodromal phase of PD using VOSviewer software (Fig. 4). The top 15 institutions are distributed across 5 countries, including the United Kingdom, United States, Germany, Canada, and Spain; among them, 5 are located in the United Kingdom and 4 in the United States. Notably close collaborations exist between the University of Tubingen and McGill University as well as between UCL and the University of Toronto.

**Table 2:** Top 15 productive institutions.

Ranking	Institution	Counts	Citations	Percentage	Total link strength	Countries/regions	Centrality
1	University of Tubingen	45	1196	4.95	66	Germany	0.02
2	McGill University	33	2615	3.63	98	Canada	0.03
3	University of London	33	2061	3.63	37	United Kingdom	0.02
4	University of Toronto	25	3751	2.75	64	Canada	0.1
5	Columbia University	23	731	2.53	55	United States	0.07
6	Kings Coll London	23	943	2.53	49	United Kingdom	0.01
7	University of Pennsylvania	23	993	2.53	48	United States	0.01
8	Newcastle University	21	1699	2.31	40	United Kingdom	0.03
9	University of Barcelona	20	1091	2.200	37	Spain	0.02
10	University of Oxford	19	533	2.09	32	United Kingdom	0.07
11	Hosp Clin Barcelona	19	1610	2.09	29	Spain	0.01
12	University of Cambridge	19	972	2.09	26	United Kingdom	0.02
13	University of Kiel	18	1690	1.98	58	Germany	0.03
14	Harvard Med Sch	18	1454	1.98	51	United States	0.02
15	Massachusetts Gen Hosp	18	1193	1.98	49	United States	0.01

### 3.4. Journal analysis

**Figure 5.**
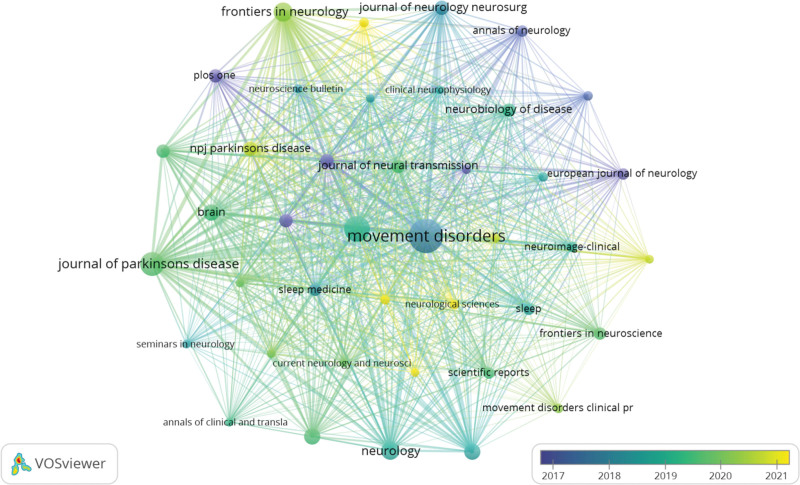
Cooperative network of journal analysis.

The prodromal phase of PD was the subject of publications in a total of 273 journals. Among these, the top 15 high-yielding journals are listed in Table 3. MOVEMENT DISORDERS emerged as the journal with the highest number of papers published (n = 98, 10.8%), followed by PARKINSONISM & RELATED DISORDERS (n = 53, 5.8%), JOURNAL OF PARKINSONS DISEASE (n = 44, 4.8%), and FRONTIERS IN NEUROLOGY (n = 29, 3.2%). According to Journal Citation Reports (2022), these major journals have an average impact factor (IF) of 9.99. The highest IF among these top 15 journals is found in LANCET NEUROLOGY (IF = 48), followed by BRAIN (IF = 14.5). Subsequently, we identified and screened a total of 41 relevant journals based on a minimum publication threshold equal to or greater than 5 articles each, resulting in the creation of a journal cooperative network using VOSviewer(Fig. 5). Figure 5 illustrates that MOVEMENT DISORDERS exhibits active reference relationships with PARKINSONISM & RELATED DISORDERS, JOURNAL OF PARKINSONS DISEASE, and other related publications.

**Table 3:** Top 15 productive journals.

Ranking	Journal	Counts	Percentage	Citations	H-index	IF (2022)	Quartile in category
1	MOVEMENT DISORDERS	98	10.78	5420	39	8.6	Q1
2	PARKINSONISM & RELATED DISORDERS	53	5.83	1139	16	4.1	Q2
3	Journal of Parkinson’s Disease	44	4.84	859	13	5.2	Q1
4	Frontiers in Neurology	29	3.19	407	11	3.4	Q2
5	NEUROLOGY	25	2.75	1070	14	9.9	Q1
6	JOURNAL OF NEUROLOGY	19	2.09	482	12	6	Q1
7	BRAIN	19	2.09	1634	15	14.5	Q1
8	Frontiers in Aging Neuroscience	19	2.09	311	11	4.8	Q2
9	JOURNAL OF NEUROLOGY NEUROSURGERY AND PSYCHIATRY	16	1.76	607	11	11	Q1
10	npj Parkinsons Disease	15	1.65	94	5	8.7	Q1
11	LANCET NEUROLOGY	14	1.54	3812	14	48	Q1
12	JOURNAL OF NEURAL TRANSMISSION	14	1.54	413	8	3.3	Q2
13	NEUROBIOLOGY OF DISEASE	14	1.54	427	9	6.1	Q1
14	Translational Neurodegeneration	13	1.43	300	9	12.6	Q1
15	PLoS One	13	1.43	570	9	3.7	Q2

IF = impact factor.

### 3.5. Co-cited journals

**Figure 6.**
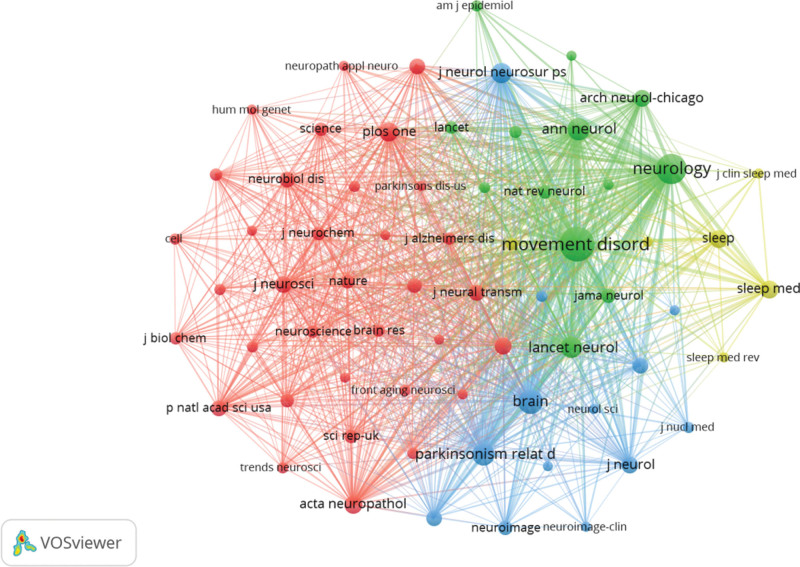
Cooperative network of Co-cited Journal analysis.

Co-citation refers to the phenomenon in which multiple authors or their articles are cited simultaneously by other scholarly literature. As depicted in Tables 4 and 8, among the top 15 highly cited journals, more than 1000 citations were attributed to MOVEMENT DISORDERS (co-citation = 7035), making it the most frequently cited journal. NEUROLOGY followed suit with a co-citation count of 4447, while BRAIN and PARKINSONISM & RELATED DISORDERS garnered co-citations of 2326 and 1908, respectively. Furthermore, LANCET NEUROLOGY boasted the highest IF (IF = 48), closely trailed by BRAIN (IF = 14.5). We identified and selected a total of 66 journals meeting our minimum citation threshold of 150 for further analysis, subsequently constructing a journal network map using VOSviewer (Fig. 6).

**Table 4:** Top 15 productive Co-cited Journal.

Ranking	Co-cited Journal	Citation frequency	Centrality	IF (2022)	Quartile in category	Total link strength
1	MOVEMENT DISORDERS	7035	0.00	8.6	Q1	418930
2	NEUROLOGY	4447	0.00	9.9	Q1	298532
3	BRAIN	2326	0.01	14.5	Q1	176508
4	PARKINSONISM & RELATED DISORDERS	1908	0.00	4.1	Q2	151829
5	ANNALS OF NEUROLOGY	1762	0.01	11.2	Q1	135674
6	LANCET NEUROLOGY	1662	0.00	48	Q1	114580
7	JOURNAL OF NEUROLOGY NEUROSURGERY AND PSYCHIATRY	1382	0.00	11	Q1	101110
8	PLoS One	1045	0.01	3.7	Q2	78834
9	ACTA NEUROPATHOLOGICA	893	0.04	12.7	Q1	80797
10	SLEEP	838	0.00	5.6	Q1	55099
11	SLEEP MEDICINE	834	0.00	4.8	Q1	54865
12	JOURNAL OF NEUROLOGY	796	0.01	6.0	Q1	62684
13	JOURNAL OF NEUROSCIENCE	778	0.01	5.3	Q1	70841
14	NEUROBIOLOGY OF AGING	735	0.01	4.2	Q2	60687
15	ARCHIVES OF NEUROLOGY	724	0.03	-	-	58174

IF = impact factor.

**Figure 7.**
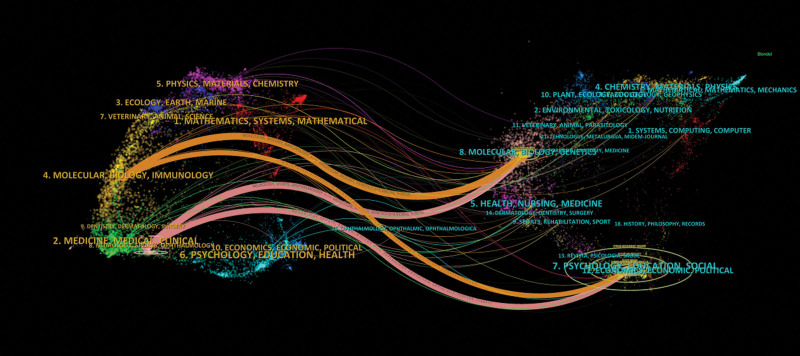
The dual-map overlay of journals.

The double graph overlay in the journal illustrates the citation relationship between the focal journal and co-cited journals, with contributing journals forming a cluster on the left and cited journals forming a cluster on the right. As depicted in Figure 7, the orange pathway represents research primarily published in MOLECULAR/BIOLOGY/GENETICS journal and referenced by literature from MOLECULAR/BIOLOGY/IMMUNOLOGY journal, while focusing on PSYCHOLOGY/EDUCATION/SOCIAL journal. Similarly, the pink pathway indicates research predominantly published in MOLECULAR/BIOLOGY/GENETICS journal and referenced by literature from NEUROLOGY/SPORTS/OPHTHALMOLOGY journal, also emphasizing PSYCHOLOGY/EDUCATION/SOCIAL aspects.

### 3.6. Contribution of authors

**Figure 8.**
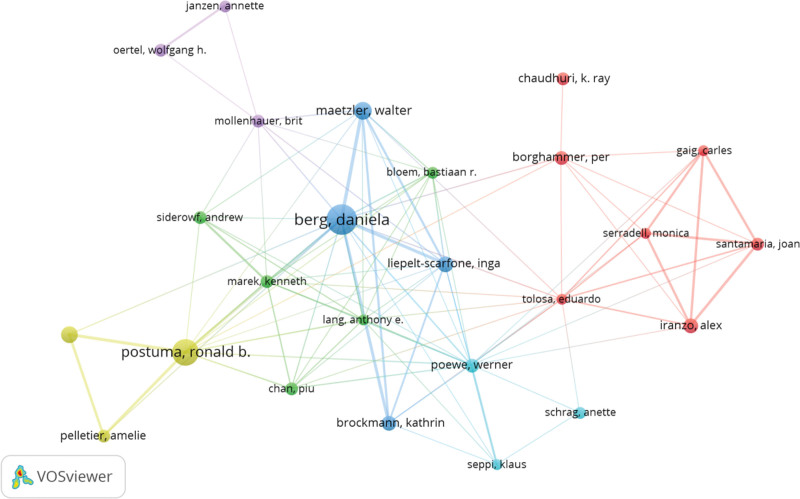
The visualization of authors.

A total of 4373 authors participated in the investigation of prodromal PD. Among the top 15 authors, three individuals each contributed to 20 publications (Table 5). To construct a collaborative network, we selected 28 authors who had published at least 10 papers (Fig. 8). Notably, Berg (n = 49; 5.39%), Postuma (n = 40; 4.40%), and Maetzler, Walter (n = 21;2.31%) emerged as key contributors with significant publication records in this field. Furthermore, our analysis revealed strong collaboration among multiple authors such as Berg closely collaborating with Maetzler Walter^[1]^ and Liepelt-Scarfone along with brockmann kathrin.^[4]^

**Table 5:** Top 15 productive authors.

Ranking	Authors	Counts	Citations	Year	Percentage	H-index	Countries/regions	Total link strength
1	Berg, Daniela	49	2857	2011	5.39	24	Germany	89
2	Postuma, Ronald B	40	2995	2012	4.4	26	Canada	57
3	Maetzler, Walter	21	622	2012	2.31	14	Germany	47
4	Gagnon, Jean-Francois	18	881	2013	1.98	15	Canada	30
5	Lliepelt-Scarfone, Inga	17	1189	2011	1.87	12	Germany	43
6	Iranzo, Alex	15	1262	2013	1.65	13	Spain	38
7	Brockmann, Kathrin	15	473	2014	1.65	12	Germany	34
8	Poewe, Werner	14	3967	2013	1.54	14	Austria	36
9	Borghammer, Per	14	894	2011	1.54	9	Denmark	9
10	Pelletier, Amelie	13	555	2013	1.43	10	Canada	27
11	Chaudhuri, K. Ray	13	322	2017	1.43	10	United Kingdom	1
12	Santamaria, Joan	12	1223	2013	1.32	12	Spain	38
13	Marek, Kenneth	12	1550	2012	1.32	11	United States	23
14	Chan, Piu	12	1180	2014	1.32	7	China	15
15	Siderowf, Andrew	12	704	2012	1.32	9	United States	15

### 3.7. Contribution of co-cited authors

**Figure 9.**
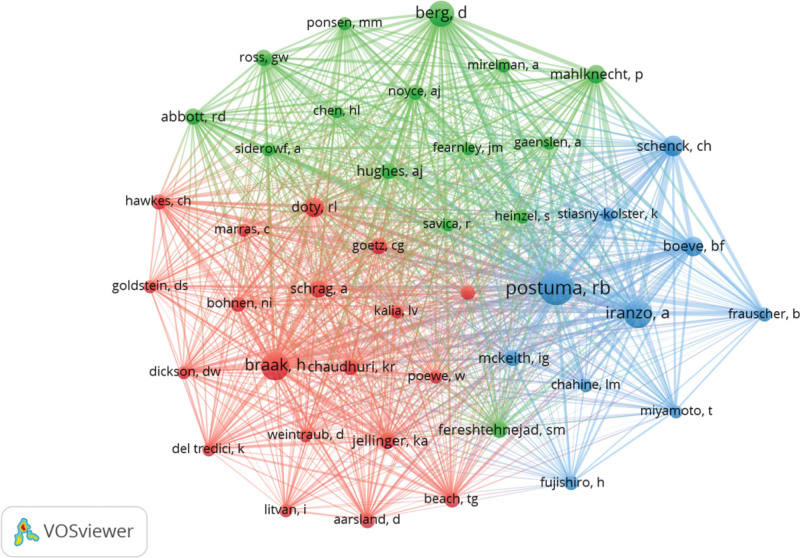
The visualization of co-cited authors.

Among the 22,579 co-cited authors, 4 were cited more than 500 times (Table 6). The most frequently cited authors are Postuma (n = 1206), followed by Iranzo, Alex (n = 667), Braak (n = 661), and Berg (n = 552). To map the co-citation network diagram, we selected 44 authors with a minimum number of co-citation times greater than or equal to 90 (Fig. 9). As shown in Figure 9, there is also active collaboration among different co-cited authors such as Postuma and Iranzo, Alex as well as Boeve, Bradley F.

**Table 6:** Top 15 co-cited authors.

Ranking	Co-cited Authors	Citation frequency	Total link strength	Countries/regions
1	Postuma, Ronald B^[5]^	1206	44311	Canada
2	Iranzo, Alex^[6]^	667	28559	Spain
3	Braak, Heiko^[7]^	661	25525	Germany
4	Berg, Daniela^[1]^	552	19908	Germany
5	Schenck, Carlos H^[8]^	287	13857	United States
6	Boeve, Bradley F^[9]^	265	13083	United States
7	Doty, Richard L^[10]^	260	10557	United States
8	Mahlknecht, Philipp^[11]^	233	10377	Austria
9	Schrag, Anette^[12]^	183	9050	United Kingdom
10	Chaudhuri, K Ray^[13]^	175	7929	United Kingdom
11	Jellinger, KA^[14]^	172	7303	Austria
12	Fereshtehnejad^[15]^	172	6965	Canada
13	McKeith, Ian G^[16]^	166	6678	United Kingdom
14	Hughes, Andrew J^[17]^	161	6648	United Kingdom
15	Abbott, Robert D^[18]^	158	6506	United States

### 3.8. Co-cited references

**Figure 10.**
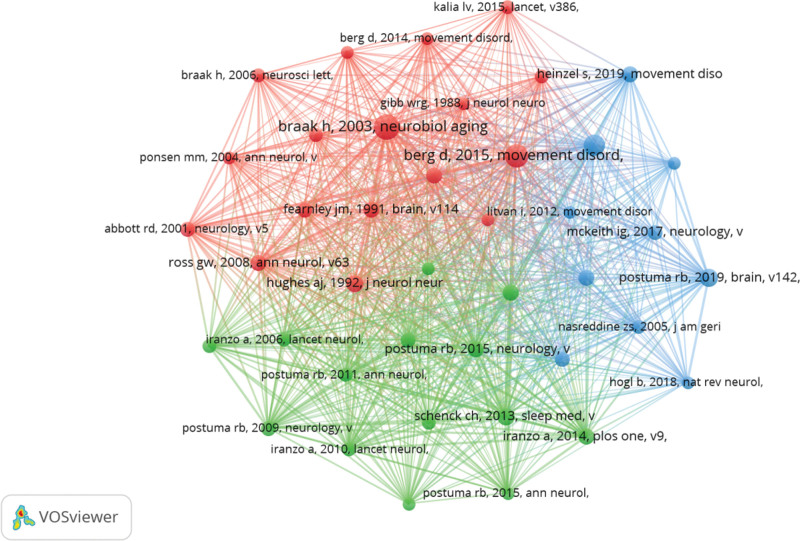
The visualization of co-cited references.

Over the past 2 decades, there have been 20,422 cited references to the prodromal phase of PD. Among the top 15 cited references (Table 7), all references were simultaneously cited at least 78 times. The highest citation (n = 275) was received by Braak’s co-cited reference entitled “Staging of brain pathology related to sporadic PD,” published in Neurobiol Aging in 2003. This paper investigated 3 groups of cases: the first group comprised brains obtained at autopsy from 41 individuals clinically diagnosed with PD; the second group included autopsy brain composition from 69 people; and the third group consisted of age- and sex-matched cases totaling 58 individuals.^[19]^ By tracing the pathologic course of episodic and symptomatic PD cases, a staging procedure based on an easily identifiable lesion terrain range is proposed. Out of the 15 references listed in Table 7, 9 were observational studies, 5 were clinical guideline studies, and 1 was a case–control study. We selected 41 references with a total citation count greater than or equal to 50 for constructing the co-citation network diagram (Fig. 10).

**Table 7:** Top 15 co-cited references.

Ranking	Co-cited reference	Author and publication year	Citations	Journal IF (2022)	Quartile	Research type	Journal	DOI
1	Staging of brain pathology related to sporadic Parkinson’s disease^[19]^	Braak, Heiko, 2003	275	4.2	Q2	Observational studies	MOVEMENT DISORDERS	0.1016/s0197-4580(02)00065-9.
2	MDS research criteria for prodromal Parkinson’s disease^[1]^	Berg, Daniela, 2015	215	8.6	Q1	Clinical guideline	MOVEMENT DISORDERS.	10.1002/mds.26431.
3	MDS clinical diagnostic criteria for Parkinson’s disease^[20]^	Postuma, Ronald B, 2015	170	8.6	Q1	Clinical guideline	MOVEMENT DISORDERS	10.1002/mds.26424.
4	Risk and predictors of dementia and parkinsonism in idiopathic REM sleep behavior disorder: a multicentre study^[21]^	Postuma, Ronald B, 2019	114	14.5	Q1	Observational studies	Brain.	10.1093/brain/awz030.
5	Accuracy of clinical diagnosis of idiopathic Parkinson’s disease: a clinico-pathological study of 100 cases^[22]^	Hughes, Andrew J, 1992	109	11.1	Q1	Observational studies	JOURNAL OF NEUROLOGY NEUROSURGERY AND PSYCHIATRY	10.1136/jnnp.55.3.181.
6	Delayed emergence of a Parkinsonian disorder or dementia in 81% of older men initially diagnosed with idiopathic rapid eye movement sleep behavior disorder: a 16-year update on a previously reported series^[23]^	Schenck, Carlos H, 2013	98	4.8	Q1	Observational studies	SLEEP MEDICINE	10.1016/j.sleep.2012.10.009.
7	Movement Disorder Society-sponsored revision of the Unified Parkinson’s Disease Rating Scale (MDS-UPDRS): scale presentation and clinimetric testing results^[24]^	Goetz Christopher G, 2008	95	8.6	Q1	Clinical guideline	MOVEMENT DISORDERS	10.1002/mds.22340.
8	Ageing and Parkinson’s disease: substantia nigra regional selectivity^[25]^	Fearnley J M, 1991	94	14.5	Q1	Observational studies	Brain	10.1093/brain/114.5.2283.
9	Prediagnostic presentations of Parkinson’s disease in primary care: a case-control study^[26]^	Schrag, Anette, 2015	93	48.0	Q1	Case–control study	LANCET NEUROLOGY.	10.1016/S1474-4422(14)70287-X
10	Neurodegenerative disease status and postmortem pathology in idiopathic rapid-eye-movement sleep behavior disorder: an observational cohort study^[6]^	Iranzo, Alex,2013	90	48.0	Q1	Observational studies	LANCET NEUROLOGY.	10.1016/S1474-4422(13)70056-5.
11	Update of the MDS research criteria for prodromal Parkinson’s disease^[27]^	Heinzel et al, 2019	88	8.6	Q1	Clinical guideline	MOVEMENT DISORDERS	10.1002/mds.27802.
12	Parkinson risk in idiopathic REM sleep behavior disorder: preparing for neuroprotective trials^[28]^	Postuma, Ronald B,2015	87	10.1	Q1	Observational studies	NEUROPATHOLOGICA	10.1212/WNL.0000000000001364
13	Neurodegenerative disorder risk in idiopathic REM sleep behavior disorder: study in 174 patients^[29]^	Iranzo, Alex, 2014	87	3.7	Q2	Observational studies	PLoS One	10.1371/journal.pone.0089741
14	Association of olfactory dysfunction with risk for future Parkinson’s disease^[30]^	Ross G Webster, 2008	87	11.2	Q1	Observational studies	ANNALS OF NEUROLOGY.	10.1002/ana.21291
15	The REM sleep behavior disorder screening questionnaire--a new diagnostic instrument^[31]^	Stiasny-Kolster Karin, 2007	78	8.6	Q1	Clinical guideline	MOVEMENT DISORDERS	10.1002/mds.21740

IF = impact factor; REM = rapid eye movement.

### 3.9. Reference with citation bursts

**Figure 11.**
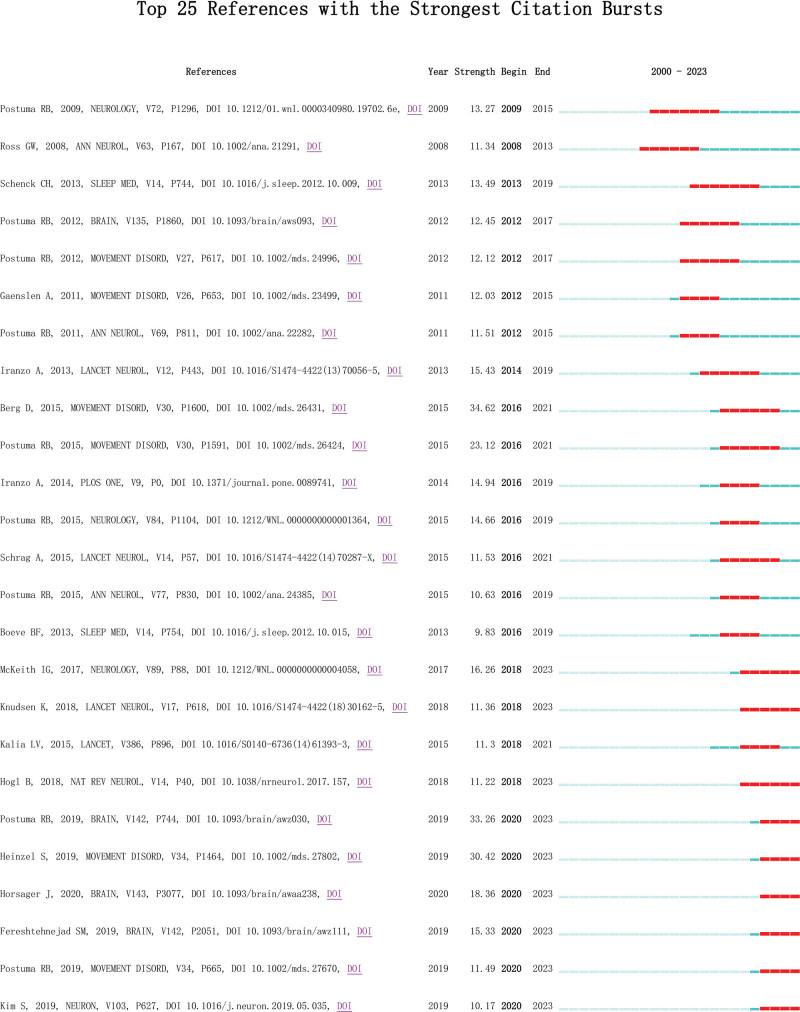
Top 25 references with strong citation bursts. A red bar indicates high citations in that year.

Citation bursts refer to references that are frequently cited by scholars in a specific field over an extended period of time. In our study, CiteSpace identified 25 references with significant citation bursts (Fig. 11). Figure 11 illustrates each bar representing a year, with the red bar indicating a strong reference burst.^[32]^ The intensity of these bursts ranged from 9.83 to 34.62, lasting between 3–6 years. These reference bursts were observed as early as 2008 and as late as 2020. The most prominent burst (intensity = 34.62) was associated with the article titled “MDS research criteria for prodromal PD” authored by Berg et al, published in MOVEMENT DISORD and cited extensively from 2016 to 2021. The second strongest burst (intensity = 33.26) was linked to the article titled “Risk and predictors of dementia and parkinsonism in idiopathic rapid eye movement (REM) sleep behavior disorder: a multicentre study” authored by Postuma et al, published in BRAIN and cited significantly from 2020 to 2023. Table 8 provides a summary of the main research contents of these referenced articles based on their sequence shown in Figure 11.

**Table 8:** The main research contents of the 25 references with strong citation bursts.

Rank	Strength	Main research content
1	13.27	The risk of neurodegenerative disease in idiopathic REM sleep behavior disorder.^[33]^
2	11.34	Association of olfactory dysfunction with risk for future Parkinson’s disease.^[30]^
3	13.49	Study of idiopathic REM sleep behavior disorder leading to Parkinson’s syndrome/dementia in middle-aged and elderly people.^[23]^
4	12.45	Prodromal parkinsonism motor changes in idiopathic REM sleep behavior disorder.^[34]^
5	12.12	Pre-motor disorders in prodromal Parkinson’s disease.^[35]^
6	12.03	To evaluate the time span from the onset of first prodromal symptoms to the initial diagnosis of PD as well as the order of symptom occurrence.^[36]^
7	11.51	Olfaction and color vision identify impending neurodegeneration in rapid eye movement sleep behavior disorder.^[37]^
8	15.43	Neurodegenerative disease status and postmortem pathology in idiopathic rapid-eye-movement sleep behavior disorder.^[6]^
9	34.62	MDS research criteria for prodromal Parkinson’s disease.^[1]^
10	23.12	Clinical diagnostic criteria for Parkinson’s disease.^[20]^
11	14.94	Neurodegenerative disorder risk in idiopathic REM sleep behavior disorder.^[29]^
12	14.66	Parkinson risk in idiopathic REM sleep behavior disorder.^[28]^
13	11.53	Prediagnostic presentations of Parkinson’s disease in primary care.^[26]^
14	10.63	Risk factors for neurodegeneration in idiopathic rapid eye movement sleep behavior disorder.^[38]^
15	9.83	Correlation of REM sleep behavior disorders with neurodegenerative lesions.^[39]^
16	16.26	Diagnosis and management of dementia with Lewy bodies.^[40]^
17	11.36	Correlation of REM sleep behavior disturbances with pathological changes in Parkinson’s disease.^[41]^
18	11.3	Advances in Parkinson’s disease.^[42]^
19	11.22	Idiopathic REM sleep behavior disorder and neurodegeneration.^[43]^
20	33.26	Risk and predictors of dementia and parkinsonism in idiopathic REM sleep behavior disorder.^[21]^
21	30.42	Update of the MDS research criteria for prodromal Parkinson’s disease.^[27]^
22	18.36	Brain-first versus body-first Parkinson’s disease: a multimodal imaging case-control study.^[44]^
23	15.33	Evolution of prodromal Parkinson’s disease and dementia with Lewy bodies.^[15]^
24	11.49	Progress in the prodromal phase of Parkinson’s disease.^[45]^
25	10.17	Transneuronal Propagation of Pathologic α-Synuclein from the Gut to the Brain Models Parkinson’s Disease.^[46]^

MDS = Movement Disorder Society; REM = rapid eye movement.

### 3.10. Analysis of co-occurrence keywords

**Figure 12.**
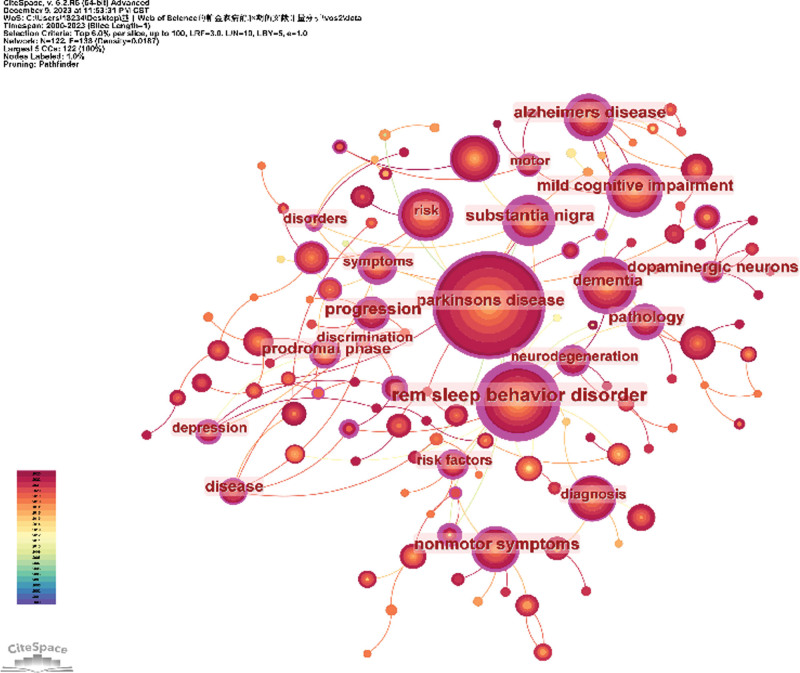
Keyword co-occurrence map.

Co-occurrence refers to the phenomenon wherein 2 or more keywords simultaneously appear in other literature. By conducting a co-occurrence analysis of keywords, one can swiftly identify research hotspots within a specific field. Table 9 presents a summary of the top 15 keywords, with PD (608 occurrences) being the most frequently utilized keyword. The subsequent prominent research directions in the prodromal phase of PD include REM sleep behavior disorder (274 cases), mild cognitive impairment (148 cases), dementia (142 cases), and risk (136 cases). Table 10 provides an overview of the top 15 keywords for centrality assessment. Keywords exhibiting high centrality signify focal points and pivotal junctures within the field, with values ranging from 0 to 1; a value ≥ 0.1 indicates substantial centrality within the domain. The 5 critical keywords with high centrality are PD (0.23), risk factors (0.15), Alzheimer’s disease (0.12), alpha-synuclein (0.1), and nonmotor symptoms(0.1), respectively, among others for this particular study scope at hand. Figure 12 illustrates a CiteSpace-generated keyword co-occurrence map pertaining to precursors of PD, encompassing a total of 369 nodes and 1408 links. The size of each node corresponds to its respective keyword frequency, while links denote co-occurrences between different keywords.

**Table 10:** Top 15 co-occurrence keyword centrality.

Cit	Count	Centrality	Year	Keywords
1	608	0.23	2000	Parkinson’s disease
2	51	0.15	2003	Risk factors
3	111	0.12	2005	Alzheimer’s disease
4	116	0.1	2009	Alpha-synuclein
5	95	0.1	2009	Nonmotor symptoms
6	57	0.1	2004	Association
7	274	0.09	2007	REM sleep behavior disorder
8	125	0.09	2008	Diagnosis
9	44	0.09	2008	Diagnostic criteria
10	38	0.09	2005	Brain
11	11	0.09	2000	Double-blind
12	14	0.08	2007	Alpha-synuclein pathology
13	148	0.07	2005	Mild cognitive impairment
14	45	0.07	2010	Features
15	30	0.07	2009	Cerebrospinal fluid

REM = rapid eye movement.

**Table 9:** Top 15 co-occurrence keyword occurrences.

Cit	Count	Centrality	Year	Keywords
1	608	0.23	2000	Parkinson’s disease
2	274	0.09	2007	REM sleep behavior disorder
3	148	0.07	2005	Mild cognitive impairment
4	142	0.06	2004	Dementia
5	136	0.06	2008	Risk
6	125	0.09	2008	Diagnosis
7	116	0.1	2009	Alpha-synuclein
8	111	0.12	2005	Alzheimer’s disease
9	95	0.1	2009	Nonmotor symptoms
10	90	0.04	2008	Substantia nigra
11	73	0.05	2008	Lewy body
12	72	0.05	2008	Dysfunction
13	68	0.02	2004	Progression
14	63	0.04	2007	Olfactory dysfunction
15	57	0.1	2004	Association

REM = rapid eye movement.

**Figure 13.**
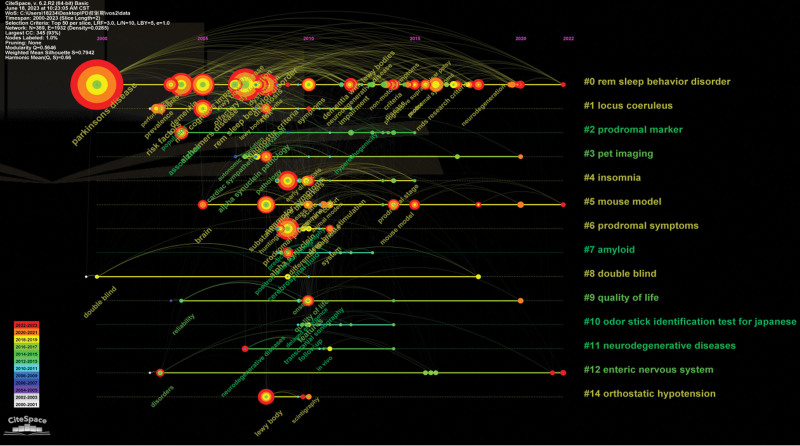
Keyword clustering analysis.

The keywords reflect the primary objectives and purposes of the research, while keyword cluster analysis represents the current hotspots and research frontiers in this field. Figure 13 presents a plot illustrating the keyword clusters for the prodromal phase of PD. This study includes terms such as “REM sleep behavior disorder,” “locus coeruleus,” “prodromal marker,” “PET imaging,” “insomnia,” “mouse model,” “prodromal symptoms,” “amyloid,” “double-blind,” “quality of life,” “odor stick identification test for Japanese individuals,” neurodegenerative diseases,” enteric nervous system,” and orthostatic hypotension.” As shown in Figure 11, nonmotor symptoms during the prodromal phase of PD have been a prominent area of research from 2000 to 2023. These include REM sleep behavior disorder, insomnia, olfaction disorders, and orthostatic hypotension. Additionally, there is significant interest in identifying biological markers during this phase through animal experiments and randomized controlled clinical trials using popular detection methods like positron emission tomography imaging. Research on tissues such as locus coeruleus and enteric nervous system has also gained attention.

## 4. Discussion

### 4.1. General information

From 2000 to 2007, 9 papers were published, with 1.5 papers published yearly. At this time, research works on the prodromal stage of PD, which were still in the conceptual stage, were few. From 2008 to 2016, 174 papers were published, with an average annual publication of 19.3 papers, which is in the initial stage of research. From 2017 to 2022, 685 papers were published, with an average annual publication of 114.2 papers, which is in the stage of rapid development of research. In the past 6 years, the number of published papers has increased significantly, indicating that the research on the prodromal phase of PD is in a period of rapid outbreak, which is a hot topic for many scholars.

The United States, Germany, and the United Kingdom were the main countries for PD prodromal research, with the United States leading the list of publications (n = 265, 29.15%). Approximately 5 of the top 15 research institutions are based in the UK and 4 in the United States. The United States, Germany, Canada, and the United Kingdom work closely together in terms of research institutions, and a good partnership between the University of Tubingen and McGill University, UCL, and the University of Toronto has been noted.

Most research studies on the prodromal phase of PD are published in Movement Disorders (IF = 8.6, Q1), which is currently the most popular journal in this field with the highest IF (IF = 48, Q1), followed by Brain (IF = 14.5, Q1). Regarding co-cited journals, most of them are high-impact Q1 journals. These journals are clearly of high quality and provide support for research in the prodromal phase of PD. Moreover, the current research on the prodromal phase of PD is mainly published in journals related to PD and neurology. Berg (n = 49, 5.39%), Postuma (n = 40, 4.40%), and Walter Maetzler (n = 21, 2.31%) published the most articles.

In 2017, Postuma summarized the research progress in the prodromal phase of PD as the potential molecular pathogenesis involving multiple pathways and mechanisms as follows^[7]^: α-synuclein protein homeostasis, mitochondrial function, oxidative stress, calcium homeostasis, axonal transport, and neuroinflammation. Recent studies of diagnostic biomarkers utilized neuroimaging, where multiple modalities, including positron emission tomography, single photon emission computed tomography, and novel magnetic resonance imaging techniques have been demonstrated to aid in early and differential diagnoses. PD treatment is based on drug replacement of striatal dopamine, in addition to non-DA approaches to address motor and nonmotor symptoms and motor complications associated with deep brain stimulation of levodopa in those with intractable PD.

In 2016, Alberto et al^[47]^ evaluated the environmental and behavioral factors that alter the risk of PD through multiple longitudinal studies. An increased risk of PD was associated with exposure to pesticides, consumption of dairy products, history of melanoma, and traumatic brain injury, whereas a reduced risk of PD was associated with smoking, caffeine intake, higher serum urate concentrations, physical activity, and the use of ibuprofen and other common medications. With these findings, identifying PD in its prodromal stage and facilitating neuroprotective interventions before motor symptoms appear may be possible in the future.

### 4.2. Knowledge base

Co-citation references refer to references cited together by multiple publications and can be considered the basis for research in one field. In this bibliometric study, we selected the 15 most co-cited references for determining the research progress in the prodromal phase of PD. Braak et al^[19]^ published the most cited study in 2003 (n = 275) in 3 groups of cases: the first group comprised brains obtained at autopsy from 41 individuals clinically diagnosed with PD; the second group comprised autopsy brains from 69 individuals; and the third group comprised 58 age- and sex-eligible cases. By tracing the pathologic course of episodic and symptomatic PD cases, a staging procedure based on an easily identifiable lesion terrain range is proposed. Overall, the top 15 co-cited references on the prodromal phase of PD focused on the following topics: pathological staging, staging criteria, and nonmotor symptoms. Among them, 3 were studies on pathological stage, 4 were on stage standard, and 8 were on nonmotor symptoms. Six of them were studies on REM sleep behavior disorder. Postuma published 3 papers out of these 15 total citations, the first of which set the criteria for the clinical diagnosis of PD, and the second and third papers are studies on the risk of neurodegeneration caused by REM sleep behavior disorder. Meanwhile, Alex Iranzo published 2 of these 15 coauthored papers, both of which are studies of REM sleep behavior disorders on the risk of neurodegeneration.

### 4.3. Hotspots and frontiers

References with citation bursts represent emerging topics within a particular field of study because they have been frequently cited by researchers in recent years. According to the main research content of the high-trigger literature (Table 8), the pathological mechanism, staging diagnostic criteria, and nonmotor symptoms of prodromal PD are the main topics of the current research. The nonmotor manifestations observed in prodromal PD encompass REM sleep behavior disorder, olfactory dysfunction, and cognitive impairment, among others. Berg in the article “MDS research criteria for prodromal PD” published in Movement Disorders in 2015 (with the highest citation intensity of 34.62, bursts time from 2016 to 2021), introduced the research criteria and probabilistic methods for diagnosing prodromal PD.^[1]^ Postuma’s article titled “Risk and predictors of dementia and parkinsonism in idiopathic REM sleep behavior disorder: a multicentre study” published in Brain in 2019 (citation strength 33.26, burst time from 2020 to present) assessed neurodegenerative disease risk and neurodegenerative predictors of disease in a large multicenter iRBD cohort.^[20]^ Heinzel’s article titled “Update of the MDS research criteria for prodromal PD” published in Movement Disorders in 2019 (citation strength 30.42, burst time from 2020 to present), reported an increased positive likelihood ratio for anosmia, increased new levels of diagnostic certainty for neurogenic and symptomatic orthostatic hypotension, and decreased positive likelihood ratio for hyperechogenes in the substantia nigra.^[27]^ Diabetes in men, overall cognitive deficits, physical inactivity, and low plasma uric acid levels as new marker inclusion criteria may improve the diagnostic accuracy of prodromal PD in the future. Horsager’s article titled “Brain-first versus body-first PD: a multimodal imaging case–control study” published in Brain in 2020 (citation intensity 18.36, burst time from 2020 to present) used multimodal imaging to test PD abnormal neurons α-syn and determined 2 subtypes: the origin of the brain first (α-synuclein appeared in the brain and then spread to the surrounding autonomic nervous system) or body priority (α-syn originated in the intestinal or surrounding autonomic nervous system, and then spread to the brain).^[44]^ Fereshtehnejad’s 2019 article in Brain titled “Evolution of prodromal PD and dementia with Lewy bodies: a prospective study” (citation strength 15.33, burst time from 2020 to present) followed the evolution of early motor and nonmotor performance in the prodromal phase of PD and recorded the evolution of prodromal manifestations similar to those predicted by the pathological staging model. The predicted precursor interval is up to 20 years.^[15]^ This demonstrates that the effects of pathological mechanisms, staging diagnostic criteria, and nonmotor symptoms (REM sleep behavior disorder and dementia) are current research trends and hotspots in the prodromal period of PD.

In addition to citation burst references, keywords can also help us quickly capture the distribution and evolution of hot topics in the field of PD prodromal research. From 2000 to 2023, the keywords of high frequency include PD (608), REM sleep behavior disorder (274), mild cognitive impairment (148), dementia (142), and risk (136). Keyword clustering is forming a network cluster of keywords with similar topics in the research field and displays the keywords in the cluster according to the time distribution through the timeline view to understand the evolution and stage characteristics of the keyword hotspots in the field. In this study, “REM sleep behavior disorder,” “locus coeruleus,” “prodromal marker,” “pet imaging,” “insomnia,” and “mouse” obtained “model,” “prodromal symptoms,” “amyloid,” “double blind,” “quality of life,” “odor stick identification test” for 15 key words including Japanese, neurodegenerative diseases, enteric nervous system, and orthostatic hypotension.

This study summarizes the research hotspots in 3 areas: pathogenesis, diagnostic criteria, and nonmotor symptoms of prodromal PD.

For pathogenesis, Braak et al^[19]^ analyzed 41 cadaveric brains of PD, 69 elderly cadaveric brains, and 58 clinical cases; summarized the pathological process of episodic PD and symptomatic PD cases; and proposed a range of lesions based on easy identification. Brain stem lesions in mildly affected cases are always limited to the medulla oblongata and tegmentum pontis. In stages 1 and 2, the involvement is actually limited to the medulla oblongata. In moderately involved cases (stages 3 and 4), the involvement was mainly confined to the lower and upper brainstem, without cortical lesions (stage 3) or initial involvement of the anterior medial temporal cortex (stage 4). All cases with severe pathology (most of which were clinically diagnosed PD, stages 5 and 6) had severe brain involvement, including neocortex regions.^[19]^ Kim et al^[46]^ tested the hypothesis that Braak-alpha-synuclein (α-syn) could be transmitted from the gut to the brain through the vagus nerve in animal experiments, and pathological α-syn preformed fibrils were injected into the duodenum and pyloric muscle layer.^[46]^ The spread of pathological α-syn in the brain was observed first in the dorsal motor nucleus, then in the tail of the back brain, including the locus ileus, and finally in the basolateral amygdala, nucleus raphe dorsalis, and pars compacta substantia nigra. In addition, loss of DA neurons and motor and nonmotor symptoms were observed in a similar temporal manner. Vagotomy and α-synuclein deficiency can stop the spread of α-synuclein disease from the gut to the brain and the associated neurodegeneration and behavioral deficits. This study supports the Block hypothesis in the etiology of idiopathic PD.

Regarding diagnostic criteria, in 2015, the International Parkinson and MDS published the diagnostic method for prodromal PD.^[1]^ The method uses a Bayesian naive classifier to calculate an individual’s risk of developing precursor PD based on age, diagnostic information, and background risk, where probable precursor PD is defined at 80% certainty. In 2019, the International Parkinson and MDS updated the research criteria for prodromal PD.^[21]^ Increased positive likelihood ratio for anosmia, decreased positive likelihood ratio for echinophores in the substantia nigra, increased diagnostic certainty for neurogenic and symptomatic orthostatic hypotension, diabetes in men, overall cognitive deficits, and low plasma uric acid levels are also new markers. These updates may improve the diagnostic accuracy for prodromal PD.

Regarding the nonmotor symptoms of prodromal PD, through a cohort study, Postuma et al assessed patients with idiopathic REM sleep behavior disorder using several quantitative movement measurements yearly, identified patients with PD, and matched them with normal controls according to age.^[23]^ They plotted the results of exercise tests from previous years and then evaluated them through a regression analysis to determine when the markers first deviated from normal. The sensitivity and specificity of quantitative motor markers for the diagnosis of precursors to PD were evaluated. Of the 78 patients, 20 had PD. In the regression analysis, the Unified PD Rating Scale first intersected normal values at approximately 4.5 years before diagnosis. Through direct prospective evaluation of the idiopathic REM sleep behavior disorder cohort during the phenotypic transition, recording the evolution of prodromal performance was similar to the pathological staging model prediction by Fereshtehnejad. Based on the analysis, anoia occurs first in nonmotor symptoms and is expected to occur 20 years before phenotypic transformation.^[15]^ Impaired color vision, constipation, and erectile dysfunction occur 10–16 years before phenotypic transition. Mild voiding dysfunction and subtle cognitive decline occurred 7–9 years before phenotypic transition. Among the motor symptoms, altered changes, bed turning, walking, salivation, speech, and facial expressions were disturbed from 7 to 11 years before the diagnosis of PD but remained mild until shortly before the phenotypic shift. Motor examination abnormalities began 5–7 years before phenotype switching, with the longest interval (8 years before phenotype switching). Among the main motor phenotypes, bradykinesia first appeared 5–6 years before the phenotypic shift, followed by rigidity (year 3) and tremor (year 2).

### 4.4. Advantages and shortcomings

This study had several unique strengths. First, we presented the first systematic analysis of PD precursor studies using bibliometrics to provide comprehensive guidance for scholars focusing on related research. Second, we used 2 bibliometric tools, VOSviewer and CiteSpace; therefore, our data analysis process is likely objective. Finally, bibliometric analysis provides more comprehensive hotspots and cutting-edge insights than traditional reviews. Nevertheless, this study has some limitations. First, the data in this study were obtained only from the Web of Science Core Collection database, and some relevant studies may have been missed. Second, we filtered out studies published in English, which may have underestimated the number of non-English papers. Furthermore, 2023 publications were excluded because of insufficient data.

## 5. Conclusion

Studying the prodromal stages of PD has important research value and application prospects for the prevention and treatment of PD. This rapid increase in publications indicates that PD precursors are gaining increasing attention from researchers worldwide. This study summarized the current status, hotspots, and trends in the prodromal phase of PD. The current status of research indicates that the field is in a stage of rapid development, and enhanced cooperation between countries/regions, institutions, and scholars is required to promote research progress. Research has focused on the pathogenesis, diagnostic criteria, and nonmotor symptoms of the prodromal phase of PD. However, only a few animal and clinical experiments have been conducted, and further experimental studies are required.

## Author contributions

**Conceptualization:** Shun Wang.

**Data curation:** Ning An.

**Methodology:** Ning An, Yulin Wang.

**Project administration:** Yuan Li.

**Supervision:** Hailong Li, Yan Bai.

**Visualization:** Ning An.

**Writing—original draft:** Shun Wang, Ning An, Yulin Wang, Yuan Li, Hailong Li, Yan Bai

**Writing—review & editing:** Shun Wang, Yan Bai
